# Development and validation of a risk prediction model for demoralization syndrome in patients with type 2 diabetes

**DOI:** 10.3389/fpubh.2026.1842877

**Published:** 2026-07-09

**Authors:** Miaomiao Li, Xiang Wang, Guiyue Ma, Yahui Meng, Haiyan Fang, Yukuan Miao

**Affiliations:** 1 Key Laboratory of Geriatric Nursing and Health, Anhui University of Chinese Medicine, Hefei, Anhui, China; 2 Emergency Intensive Care Unit, The First Affiliated Hospital of Anhui Medical University, Hefei, Anhui, China

**Keywords:** demoralization syndrome, prediction model, risk factors, type 2 diabetes mellitus, validation

## Abstract

**Background:**

Demoralization syndrome (DS), characterized by helplessness, hopelessness, and diminished self-worth, is a common but underrecognized psychological distress in chronic diseases. Type 2 diabetes mellitus (T2DM) affects over 537 million adults worldwide, yet DS remains underexplored in this population.

**Methods:**

A cross-sectional observational study using a convenience sampling method was conducted to recruit 852 patients with T2DM from a hospital in China between October 2023 and October 2024. Participants were divided into a training set (*n* = 598) and a temporal validation set (*n* = 254) based on the chronological order of the survey. Feature selection was conducted using LASSO regression. A risk prediction model was developed using multivariable logistic regression, and a nomogram was subsequently constructed. Bootstrap resampling and Decision Curve Analysis (DCA) were utilized for internal evaluation and clinical utility assessment.

**Results:**

LASSO and multivariable logistic regression identified eight independent influential factors for DS: educational level, two-hour postprandial plasma glucose, glycated hemoglobin A1c, diabetes self-management, the confrontation and avoidance dimensions of the medical coping modes questionnaire, resilience, and sleep quality. The model demonstrated excellent predictive accuracy, with an AUC of 0.898 in the training set (optimism-corrected AUC of 0.892 via Bootstrap) and 0.868 in the temporal validation set. DCA indicated a positive net clinical benefit across a wide range of threshold probabilities.

**Conclusion:**

This well-calibrated and highly discriminative model, validated temporally, offers healthcare professionals a robust and practical tool for the early identification of DS and the optimization of clinical nursing decisions in T2DM patients.

## 1 Introduction

Diabetes mellitus is a chronic non-communicable disease primarily characterized by glucose metabolism disorders, representing one of the major public health challenges that currently pose a severe threat to human health (1). According to statistics from the 2021 International Diabetes Federation (IDF) report, as many as 537 million adults worldwide suffer from diabetes. It is projected that the number of people with diabetes in China will reach approximately 164 million by 2030 (2), with T2DM accounting for more than 95% of these cases (2). Extensive research has indicated that diabetes not only compromises patients’ physical health and daily functioning but also, as a lifelong disease, presents unique challenges due to its nature. Patients are required to maintain long-term and stringent dietary control, engage in regular physical exercise, and perform continuous blood glucose monitoring (3). As the disease progresses and potentially manifests as severe complications, it significantly impacts patients’ mental health, triggering a spectrum of negative emotions, including anxiety (4), depression (5), and demoralization (6). Meanwhile, patients with diabetes comorbid with negative emotions exhibit impaired self-management. Such patients may develop resistance toward disease-related treatments, leading to decreased treatment adherence and the formation of a vicious cycle.

Demoralization Syndrome (DS) was first reported by Frank (7) to describe a persistent sense of subjective incompetence and its associated experience of hopelessness, and it was formally proposed by Clarke (8) in 2002 as the inability to cope with internal or external stressors. In addition to a generalized sense of inadequacy, patients often feel trapped by their illness and unable to look toward the future with optimism. It is primarily characterized by feelings of helplessness, hopelessness, and diminished self-worth (9). Crucially, DS must be clearly distinguished from depression. Although they frequently co-occur and share overlapping symptoms, depression is fundamentally driven by anhedonia, whereas DS is primarily triggered by subjective incompetence. Therefore, DS should be recognized as a distinct and independent clinical entity. Clinically, DS is frequently observed in patients grappling with severe somatic and chronic diseases. Globally, it affects approximately 10 to 30% of oncology and palliative care populations (10, 11). Furthermore, this distress is not confined to oncology; it is also widely observed in patients battling other chronic conditions, such as maintenance hemodialysis, post-stroke disability, and Parkinson’s disease (12). The consequences of DS are severe. It often leads to a loss of meaning in life and the development of self-harm or suicidal ideation, which significantly impedes the recovery process and shortens survival (13). Notably, evidence suggests that DS is an independent predictor of suicidal ideation, separate from clinical depression (13). Consequently, researchers have increasingly focused on this phenomenon in recent years, developing targeted intervention strategies to effectively prevent self-injury in affected patients.

Existing studies have demonstrated that DS is correlated with demographic factors such as gender, age, and educational level (14). Disease-related factors, including duration of illness and treatment modalities (15), may also influence its development. Notably, as DS is defined as a state of psychological distress, psychological factors have been confirmed to be closely linked to its occurrence. Research indicates that DS is significantly associated with anxiety and depression across various patient populations (9). For instance, a study of 473 patients with chronic diseases (16) showed that individuals with higher levels of depression face an increased risk of demoralization. Furthermore, research involving mixed cancer populations (17) confirmed the co-occurrence and independence of DS in relation to psychiatric disorders (including depression and anxiety) and suicidal ideation. Together, these findings underscore the importance of DS as a distinct construct of distress in predicting the mental health status of cancer patients.

Conversely, positive coping modes and resilience may serve as protective factors. Coping mode refers to the cognitive and behavioral strategies individuals employ to maintain psychological equilibrium when facing stressful situations (18). A study by Tang et al. (19) involving 296 cancer patients demonstrated that positive coping modes exert a protective effect against demoralization, whereas negative strategies, such as avoidance and resignation, exacerbate its severity.

Resilience, defined as a positive psychological trait, enables individuals to maintain mental health and restore functioning in the face of stress, trauma, or adversity (20). Existing research indicates a negative correlation between resilience, DS, and suicidal ideation; individuals with higher resilience can more effectively manage disease-related stress, thereby reducing feelings of hopelessness and helplessness and lowering the level of demoralization (21). Furthermore, evidence suggests that both subjective and objective social support can enhance social adaptability, providing resources and guidance for problem-solving. This helps individuals navigate stress and challenges more effectively, potentially preventing the onset of DS (22).

Recently, risk prediction models for psychological comorbidities in patients with T2DM have made significant progress, primarily targeting depression, anxiety, and diabetes distress. For instance, Yu et al. (23) constructed a depression risk model demonstrating the effectiveness of combining sociodemographic and clinical variables. Similarly, Feng et al. (24) developed predictive models for depression and anxiety in primary care settings. Furthermore, extensive epidemiological studies by Riise et al. (25) and Dalsgaard et al. (26) have highlighted the vital impact of lifestyle and metabolic outcomes on the psychological well-being of these patients. While these excellent studies provide valuable insights for psychological risk assessment, predictive models specifically tailored to Demoralization Syndrome (DS) in patients with T2DM remain relatively scarce. Current models predominantly focus on general depression or diabetes distress, placing less emphasis on comprehensive bio-psycho-social determinants (such as coping styles, social support, and disease perception) that are crucial for DS. To provide a more targeted assessment tool, this study aims to construct a specific risk prediction model for DS in T2DM patients. By integrating multi-dimensional bio-psycho-social data, we developed an intuitive risk assessment tool designed to assist healthcare professionals in the early identification of high-risk patients, thereby facilitating proactive and personalized psychological interventions.

## 2 Methods

### 2.1 Design and sample

This cross-sectional observational study employed a convenience sampling method. It is worth noting that deriving a convenience sample from a single tertiary hospital may carry a potential for selection bias. During the sampling process, researchers consecutively approached potential participants in the ward based on their order of admission. Initially, a total of 950 patients with T2DM were screened. Among them, 55 patients were excluded for not meeting the inclusion criteria, and 43 patients declined to participate. Ultimately, 852 patients provided informed consent and completed the survey, resulting in a response rate of 89.7%. The patients admitted between October 2023 and June 2024 were enrolled as the training set (*n* = 598), while those admitted from July 2024 to October 2024 were assigned to the temporal validation set (*n* = 254).

The inclusion criteria were as follows: (1) patients who met the diagnostic criteria of the Guidelines for the Prevention and Control of Type 2 Diabetes in China (2020 Edition) (2) or those previously diagnosed with T2DM and currently receiving pharmacological treatment; (2) age ≥ 18 years; (3) conscious with no communication barriers and able to cooperate with the investigators; (4) provided informed consent and participated in this study voluntarily. The exclusion criteria were: (1) patients with psychiatric disorders (e.g., schizophrenia, persistent mood disorders, or conversion disorder), as defined by the Chinese Classification of Mental Disorders, Third Edition (CCMD-3) (27); (2) patients in the acute phase of an illness or those in critical condition requiring continuous monitoring; (3) presence of severe complications, such as other major organ dysfunction and/or malignant tumors; (4) severe aphasia, or significant intellectual, auditory, or memory impairment, or other conditions precluding verbal communication.

The sample size was determined based on Kendall’s multivariate regression sample size estimation method, which suggests that the minimum sample size should be 5–10 times the number of independent variables (28). This study utilized seven survey instruments comprising a total of 62 variables. Accounting for a 20% potential dropout rate, yielding a minimum required sample size of 372 cases. A total of 598 valid cases were ultimately included in this study. Following the principle of allocating the training and validation groups at a 7:3 ratio, the final sample size for the validation group was 254 cases.

### 2.2 Data collection

Data were collected through face-to-face sessions using paper-based questionnaires. Prior to the assessment, standardized instructions were provided to participants to explain the study’s objectives and the content of the questionnaire. Participants were informed of the voluntary nature of their involvement, their right to withdraw at any time, and the guaranteed confidentiality of their personal information. Formal enrollment commenced only after obtaining signed informed consent. Furthermore, general demographic information, laboratory test results, and clinical data were retrieved from the hospital’s Electronic Medical Record (EMR) system. To ensure data quality, a complete-case analysis approach was adopted. Questionnaires with missing items or logically inconsistent responses were excluded during the initial data screening. Similarly, patients with missing critical clinical data (such as laboratory test results) in the EMR system were also excluded. Consequently, the final datasets comprised exclusively valid cases with complete information for all analyzed variables, and no statistical data imputation methods were necessary.

### 2.3 Measurement instruments

#### 2.3.1 General information questionnaire

The questionnaire consists of two primary sections:

(1) General demographic data includes sex, ethnicity, age, educational level, marital status, residence, work difficulty, household income, medical payment method, Family care methods.
(2) Clinical and medical records including: smoking and drinking history, BMI, Diabetes Course, Diabetes complications, Fasting blood glucose (FBG), 2-h postprandial blood glucose (2 h-PBG), glycated hemoglobin A1c (HbA1c), total cholesterol (TCHol), Triglycerides (TG), high-density lipoprotein cholesterol (HDLC), low-density lipoprotein cholesterol (LDLC), Homocysteine (HCY), glycated albumin (GA), Fasting insulin (FINS), Fasting C-Peptide (FCP).

#### 2.3.2 Demoralization scale-II

The Demoralization Scale-II was developed by Robinson et al. (29), was validated for the Chinese context by Ou et al. (30). This 16-item scale comprises two dimensions: “Meaning and Purpose” and “Distress and Coping.” Each item is rated on a 3-point Likert scale, with total scores ranging from 0 to 32. Higher scores indicate a greater severity of demoralization. In this study, the total Cronbach’s *α* for the DS-II was 0.901.

#### 2.3.3 Connor–Davidson resilience scale

The Connor–Davidson Resilience Scale was developed by Connor et al. (31), comprising 25 items across five dimensions: hardiness, tolerance of negative affect, positive acceptance of change, control, and spiritual influences. The Chinese version of the CD-RISC, translated and revised by Yu et al. (32), retains the original 25 items but reconfigures them into three dimensions: tenacity, optimism, and self-strength. Responses are recorded on a 5-point Likert scale, with total scores ranging from 0 to 100. Higher scores reflect a higher level of psychological resilience. In this study, the total Cronbach’s *α* for this scale was 0.91.

#### 2.3.4 Medical coping modes questionnaire

The Medical Coping Modes Questionnaire was developed by Feifel et al. (33) and adapted into Chinese by Shen et al. (34), consists of 20 items distributed across three dimensions: Confrontation, Avoidance, and Resignation. Each item is rated on a 4-point Likert scale. Each dimension is scored independently, with higher scores indicating a stronger tendency for the patient to adopt that specific coping strategy. In this study, the Cronbach’s *α* for the three dimensions were 0.69, 0.60, and 0.76, respectively.

#### 2.3.5 Social support rating scale

The Social Support Rating Scale was developed by Xiao (35), consists of 10 items divided into three dimensions: subjective support, objective support, and utilization of support. The total score ranges from 12 to 66 points, higher total scores reflect a greater degree of social support. The reported Cronbach’s *α* for this scale ranges from 0.89 to 0.94.

#### 2.3.6 Summary of diabetes self-care activities

The Summary of Diabetes Self-Care Activitiesscale was developed by Toobert et al. (36), was translated into Chinese by Wan et al. (37). The revised scale comprises 10 items categorized into four dimensions: dietary regulation, physical exercise, blood glucose self-monitoring, and foot care. Each item is scored using an 8-point Likert scale, with total scores ranging from 0 to 77. Higher scores indicate superior self-management adherence. The overall Cronbach’s *α* for this scale was 0.84.

#### 2.3.7 Pittsburgh sleep quality index

The Pittsburgh Sleep Quality Index was developed by Buysse et al. (38), is designed to evaluate the subjective sleep quality of patients over the past month. The scale comprises 18 scoring items categorized into seven dimensions: subjective sleep quality, sleep latency, sleep duration, habitual sleep efficiency, sleep disturbances, use of sleeping medication, and daytime dysfunction. Each item is scored on a scale of 0 to 3, with the total score ranging from 0 to 21, higher scores reflect poorer sleep quality. In this study, the overall Cronbach’s *α* of the scale was 0.845.

### 2.4 Data analysis

Statistical analyses were performed using R software (version 4.3.1). Normally distributed continuous variables were expressed as mean ± standard deviation and compared using independent samples *t*-test. Categorical and ordinal data were expressed as frequencies and percentages, and compared using the Chi-square test or Mann–Whitney U test, as appropriate.

To minimize the risk of overfitting and selection bias, the Least Absolute Shrinkage and Selection Operator (LASSO) regression with 10-fold cross-validation was employed to select the most predictive features from the training set. The optimal penalization parameter was determined using the 1 standard error of the minimum criteria (lambda.1se). Subsequently, the non-zero variables identified by the LASSO regression were incorporated into a multivariable logistic regression model to construct a nomogram for predicting the risk of Demoralization Syndrome in T2DM patients. Model performance was comprehensively evaluated in both the training and temporal validation sets. Discrimination was assessed using the area under the receiver operating characteristic (ROC) curve (AUC). Calibration was evaluated visually using calibration plots and statistically via the Hosmer-Lemeshow goodness-of-fit test.

Furthermore, in strict accordance with the TRIPOD guidelines, an internal validation was conducted using a 1,000-iteration Bootstrap resampling procedure to calculate the optimism-corrected AUC, thereby evaluating the potential optimism bias. Finally, Decision Curve Analysis (DCA) was performed to quantify the clinical utility and net benefit of the predictive model across a wide range of threshold probabilities. A two-sided *p* < 0.05 was considered statistically significant.

## 3 Results

### 3.1 Participant characteristics

In the training group (*n* = 598), 274 patients were identified with DS, representing a prevalence of 45.8%. Similarly, in the validation group (*n* = 254), 119 patients presented with DS, corresponding to a prevalence of 46.8%. There were no statistically significant differences between the two cohorts regarding general demographics, clinical history, or scores on relevant psychometric scales (*p* > 0.05). Detailed results are shown in Supplementary Table S1.

DS was analyzed as a binary variable, with a DS-II score ≤9 defining the non-DS group and a score > 9 defining the DS group. As shown in Table 1, the univariate analysis revealed statistically significant differences (*p* < 0.05) between the two groups, including sex, ethnicity, educational level, residence, work difficulty, medical payment method, and family care methods, Diabetes complications, 2 h PG, HbAlc, TCHoL, TG, HDLC, HCY, SSRS, SDSCA, MAMQ, CD-RISC, PSQI.

**Table 1:** Training group DS prevalence and associated covariates in T2DM patients (*n* = 598).

Variable	No demoralization syndrome (*n* = 324)	Demoralization syndrome (*n* = 274)	Statistical value	*p*
Sex [(*n*)%]			4.015^a^	0.045
Male	199 (61.4)	146 (53.3)		
Female	125 (38.6)	128 (47.4)		
Ethnicity [(*n*)%]			5.378^a^	0.020
Han majority	315 (97.2)	255 (93.1)		
Ethnic minorities	9 (2.8)	19 (6.9)		
Age [years old, (*n*)%]			0.097^a^	0.796
<45	65 (20.1)	54 (19.7)		
45–60	120 (37.0)	99 (36.1)		
>60	139 (42.9)	121 (44.2)		
Educational level [(*n*)%]			33.283^a^	<0.001
Uneducated	30 (9.3)	45 (16.4)		
Elementary school	39 (12.0)	75 (27.4)		
Junior high school	58 (17.9)	49 (17.9)		
High school	81 (25.0)	48 (17.5)		
Associate degree or higher	116 (35.8)	57 (20.8)		
Marital status [(*n*)%]			0.918^a^	0.632
Unmarried	16 (4.9)	12 (4.4)		
Married	283 (87.3)	235 (85.8)		
Divorced or widowed	25 (7.7)	27 (9.9)		
Residence [(*n*)%]			6.470^a^	0.011
Town	224 (69.1)	162 (59.1)		
Rural	100 (30.9)	112 (40.9)		
Work difficulty [(*n*)%]			3.567^a^	0.059
Easy	71 (21.9)	80 (29.2)		
Moderate	152 (46.9)	120 (43.8)		
Difficult	101 (31.2)	74 (27.0)		
*Per capita* monthly household income [(RMB), (*n*)%]			0.010^a^	0.919
<1,000	34 (10.5)	27 (9.9)		
1,000–3,000	65 (20.1)	61 (22.3)		
3,000–5,000	113 (34.9)	91 (33.2)		
>5,000	112 (34.6)	95 (34.7)		
Medical payment method [(*n*)%]			13.271^a^	0.001
Employee medical insurance	198 (61.1)	127 (46.4)		
Urban resident medical insurance	116 (35.8)	138 (50.4)		
Self-funded	10 (3.1)	9 (3.3)		
Family care methods [(*n*)%]			3.331^a^	0.068
Family involvement in management assistance	146 (45.1)	144 (52.6)		
No family involvement in management assistance	178 (54.9)	130 (47.4)		
Smoking history [(*n*)%]			0.584^a^	0.445
Yes	97 (29.9)	90 (32.8)		
No	227 (70.1)	184 (67.2)		
Drinking history [(*n*)%]			0.011^a^	0.918
Yes	91 (28.1)	78 (28.5)		
No	233 (71.9)	196 (71.5)		
BMI [Kg/m^2^, (*n*)%]			4.279^a^	0.154
<18.5	9 (2.8)	6 (2.2)		
18.5~	118 (36.4)	80 (29.2)		
24~	119 (36.7)	119 (43.4)		
>28	78 (24.1)	69 (25.2)		
Diabetes course [year, (*n*)%]			0.046^a^	0.829
<1	48 (14.8)	45 (16.4)		
1–5	73 (22.5)	62 (22.6)		
6–10	63 (19.4)	45 (16.4)		
>10	140 (43.2)	122 (44.5)		
Diabetes complications [(*n*)%]			16.829^a^	<0.001
No	172 (53.1)	105 (38.3)		
1	83 (25.6)	71 (25.9)		
2	40 (12.3)	56 (20.4)		
≥3	29 (9.0)	42 (15.3)		
FBG [mmol/L, ( $\bar{x}\pm s$ )]	8.56 ± 2.98	8.33 ± 2.55	0.957^b^	0.328
2 h PG [mmol/L, ( $\bar{x}\pm s$ )]	11.00 ± 3.12	15.73 ± 5.12	108.622^b^	<0.001
HbA1c [%, ( $\bar{x}\pm s$ )]	8.71 ± 2.43	11.40 ± 3.51	80.681^b^	<0.001
TCHoL [mmol/L, ( $\bar{x}\pm s$ )]	3.86 ± 1.88	3.36 ± 2.09	9.386^b^	0.002
TG [mmol/L, ( $\bar{x}\pm s$ )]	2.65 ± 2.32	3.29 ± 2.53	10.017^b^	0.002
HDLC [mmol/L, ( $\bar{x}\pm s$ )]	1.40 ± 0.54	1.58 ± 0.68	11.790^b^	<0.001
LDLC [mmol/L, ( $\bar{x}\pm s$ )]	3.70 ± 1.69	3.89 ± 1.87	1.637^b^	0.201
HCY [μmol/L, ( $\bar{x}\pm s$ )]	12.23 ± 5.85	13.16 ± 6.87	3.159^b^	0.075
GA [%, ( $\bar{x}\pm s$ )]	22.35 ± 7.35	21.48 ± 6.65	2.274^b^	0.132
FINS [mU/L, ( $\bar{x}\pm s$ )]	15.06 ± 7.24	14.19 ± 7.22	2.145^b^	0.143
FCP [pmol/L, ( $\bar{x}\pm s$ )]	2.73 ± 1.39	2.57 ± 1.41	1.889^b^	0.169
SSRS [score, ( $\bar{x}\pm s$ )]	26.38 ± 3.46	25.67 ± 3.85	5.471^b^	0.019
MCMQ [score, ( $\bar{x}\pm s$ )]				
Confrontation	19.90 ± 3.01	18.04 ± 3.34	43.541^b^	<0.001
Avoidance	17.13 ± 2.47	17.92 ± 2.74	13.307^b^	<0.001
Resignation	11.28 ± 2.52	12.42 ± 2.58	27.132^b^	<0.001
SDSCA [score, ( $\bar{x}\pm s$ )]	25.62 ± 8.56	20.45 ± 6.98	52.964^b^	<0.001
CD-RISC [score, ( $\bar{x}\pm s$ )]	45.55 ± 11.16	35.80 ± 5.75	105.511^b^	<0.001
PSQI [score, ( $\bar{x}\pm s$ )]	7.17 ± 3.17	9.19 ± 2.63	57.339^b^	<0.001

a *χ*^2^ value.

b *t* value.

FBG, fasting blood glucose; 2 h PG, two-hour postprandial plasma glucose; HbAlc, glycated hemoglobin A1c; TCHoL, total cholesterol; TG, triglycerides; HDLC, high-density lipoprotein cholesterol; LDLC, low-density lipoprotein cholesterol; HCY, homocysteine; GA, glycated albumin; FINS, fasting insulin; FCP, fasting C-peptide; SSRS, social support rating scale; SDSCA, summary of diabetes self-care activities. MAMQ, medical coping modes questionnaire; CD-RISC, Connor Davidson resilience scale; PSQI, Pittsburgh sleep quality index.

### 3.2 Feature selection using LASSO regression

**Figure 1:**
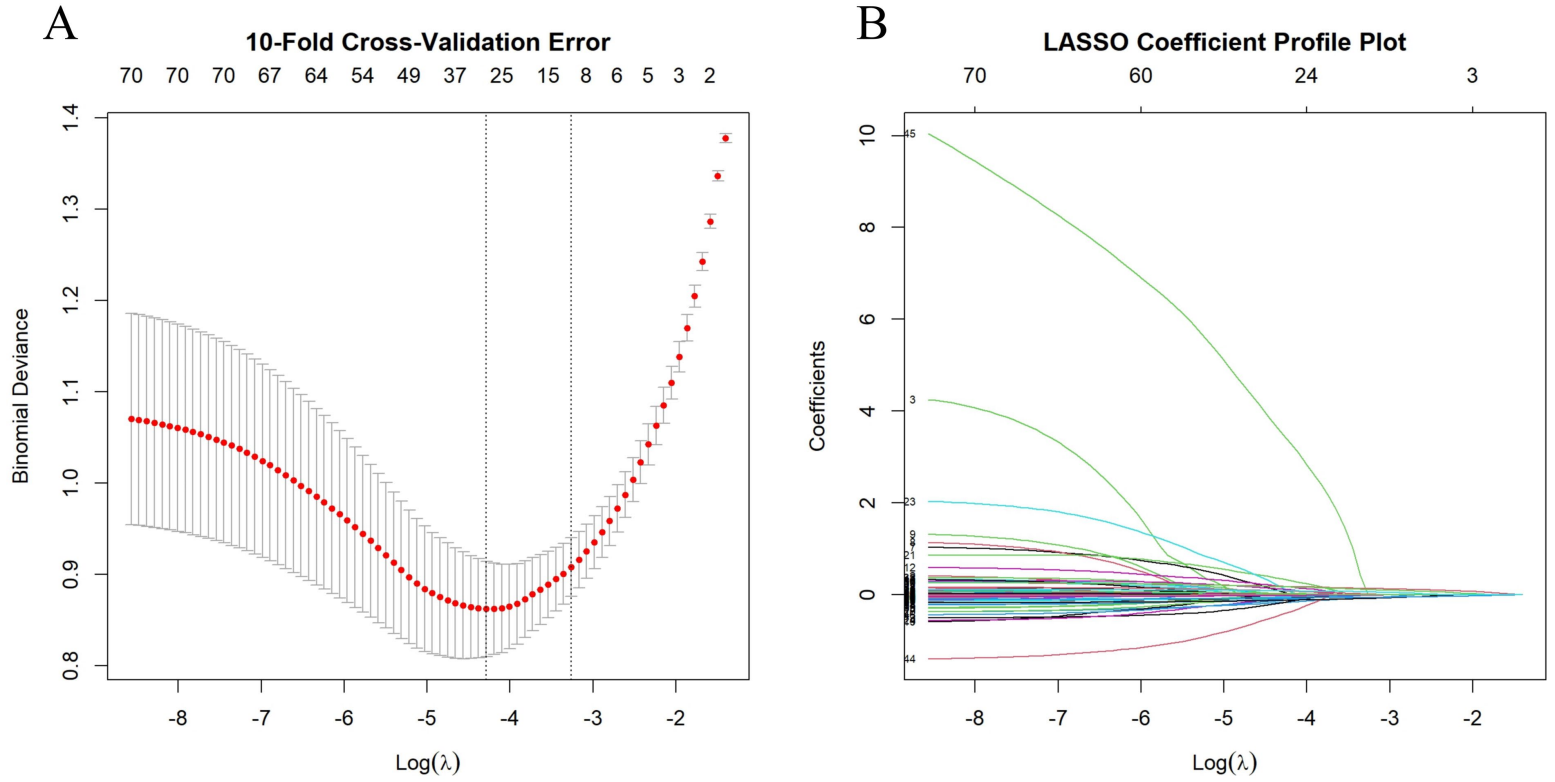
Feature selection using the LASSO regression model. **(A)** 10-fold cross-validation error plot for the LASSO model. **(B)** LASSO coefficient profiles of potential risk factors for Demoralization Syndrome in T2DM patients.

Among the 62 initial candidate variables (including demographic characteristics, clinical indices, and psychometric scores), 8 features with non-zero coefficients were ultimately selected via LASSO regression based on the optimal penalization parameter (lambda.1se; Figures 1A,B). These features included educational level, 2 h PG, HbA1c, SDSCA score, the confrontation and avoidance dimensions of the MCMQ, CD-RISC score and PSQI score (variable assignments are detailed in Supplementary Table S2).

### 3.3 Multivariable logistic regression analysis of DS in patients with T2DM

The logistic regression results are presented in Table 2. Incorporating these 8 variables into the multivariable logistic regression model revealed that 2 h PG (OR = 1.202, 95% CI: 1.139–1.275, *p* < 0.001), HbA1c (OR = 1.207, 95% CI: 1.116–1.312, *p* < 0.001), the avoidance dimension of the MCMQ (OR = 1.108, 95% CI: 1.021–1.205, *p* = 0.015), and PSQI scores (OR = 1.139, 95% CI: 1.057–1.229, *p* < 0.001) were independent risk factors for DS. Conversely, educational level (OR = 0.824, 95% CI: 0.701–0.969, *p* = 0.019), SDSCA scores (OR = 0.969, 95% CI: 0.939–0.999, *p* = 0.043), resilience scores (CD-RISC: OR = 0.919, 95% CI: 0.892–0.945, *p* < 0.001), the confrontation dimension of the MCMQ (OR = 0.854, 95% CI: 0.794–0.915, *p* < 0.001) served as independent protective factors against DS. Collinearity diagnostics revealed that all VIF values for the 8 variables in the final model were less than 5 (ranging from 1.015 to 1.088), indicating that there was no significant multicollinearity among the predictors, thereby ensuring the stability of the regression coefficients. Based on the multivariable regression coefficients, the predictive model equation for the probability of DS is as follows:

**Table 2:** Logistic regression analysis of demoralization syndrome in research subjects.

Variable	*β*	SE	Wald *χ*^2^	*p* value	OR	95%CI
Constant	0.320	1.345	0.057	0.812	–	–
Educational level	−0.193	0.082	5.490	0.019	0.824	0.701–0.969
2 h PG	0.184	0.029	41.037	<0.001	1.202	1.139–1.275
HbA1c	0.188	0.041	20.885	<0.001	1.207	1.116–1.312
SDSCA score	−0.032	0.016	4.101	0.043	0.969	0.939–0.999
MCMQ						
Confrontation	−0.158	0.036	19.158	<0.001	0.854	0.794–0.915
Avoidance	0.102	0.042	5.890	0.015	1.108	1.021–1.205
CD-RISC score	−0.084	0.015	33.178	<0.001	0.919	0.892–0.945
PSQI score	0.130	0.038	11.533	0.001	1.139	1.057–1.229

SE, standard error; OR, odds ratio; 95%CI, 95% confidence interval; 2 h PG, two-hour postprandial plasma glucose; HbAlc, glycated hemoglobin A1c; SDSCA, summary of diabetes self-care activities; MAMQ, medical coping modes questionnairen; CD-RISC, Connor Davidson resilience scale; PSQI, pittsburgh sleep quality index.

Logit(*P*) = 0.320–0.193 × Educational Level + 0.184 × 2 h PG + 0.188 × HbA1c - 0.032 × SDSCA score - 0.158 × MCMQ_Confrontation score + 0.102 × MCMQ_Avoidance score - 0.084 × CD-RISC score + 0.130 × PSQI score.

### 3.4 Development and validation of predictive models

#### 3.4.1 Model construction

**Figure 2:**
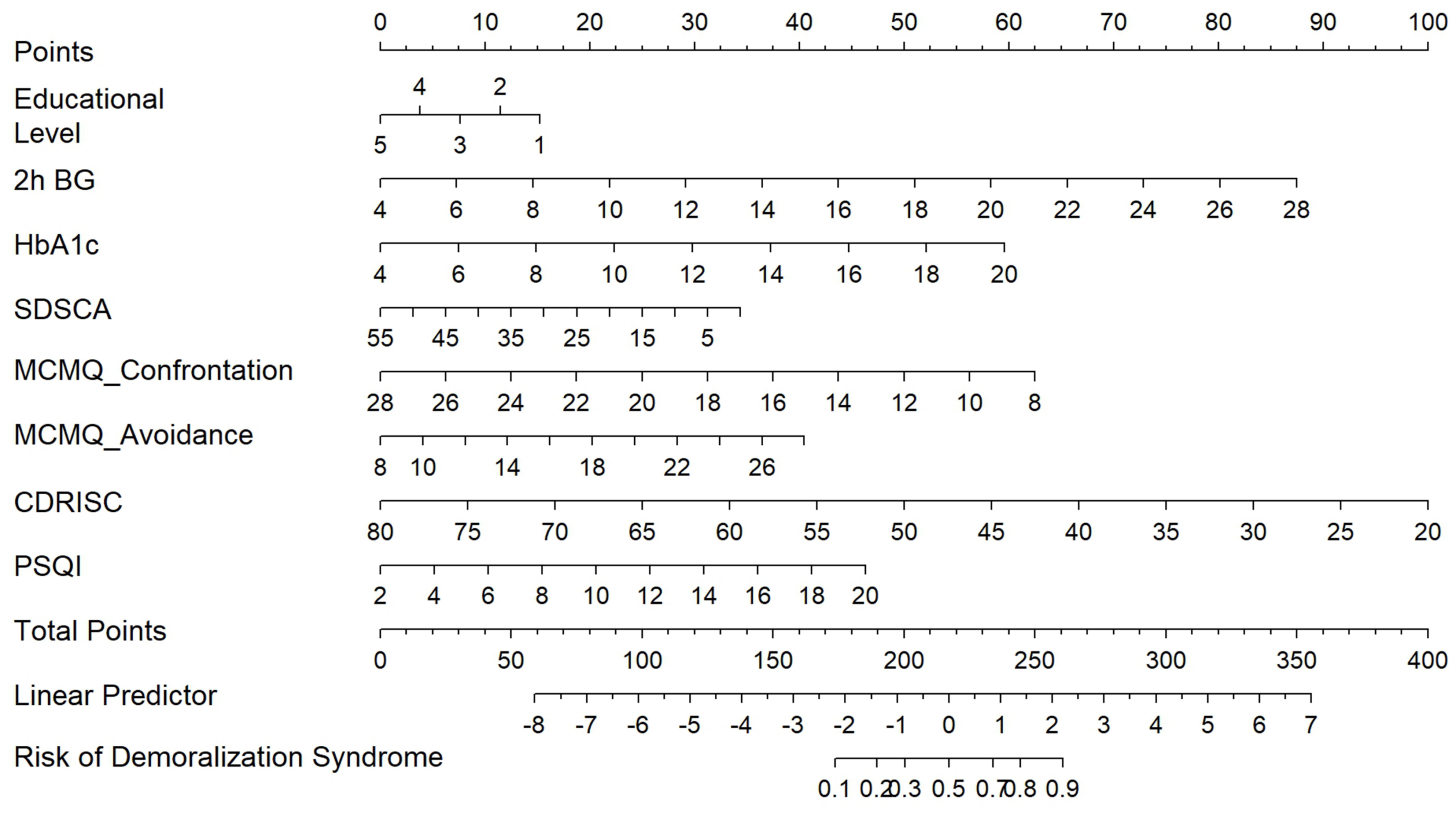
Nomogram for predicting the risk of Demoralization Syndrome (DS) in patients with T2DM.

The aforementioned multivariable logistic regression model, incorporating the 8 identified independent influential factors, was visualized using a nomogram (Figure 2).

#### 3.4.2 Internal validation

**Figure 3:**
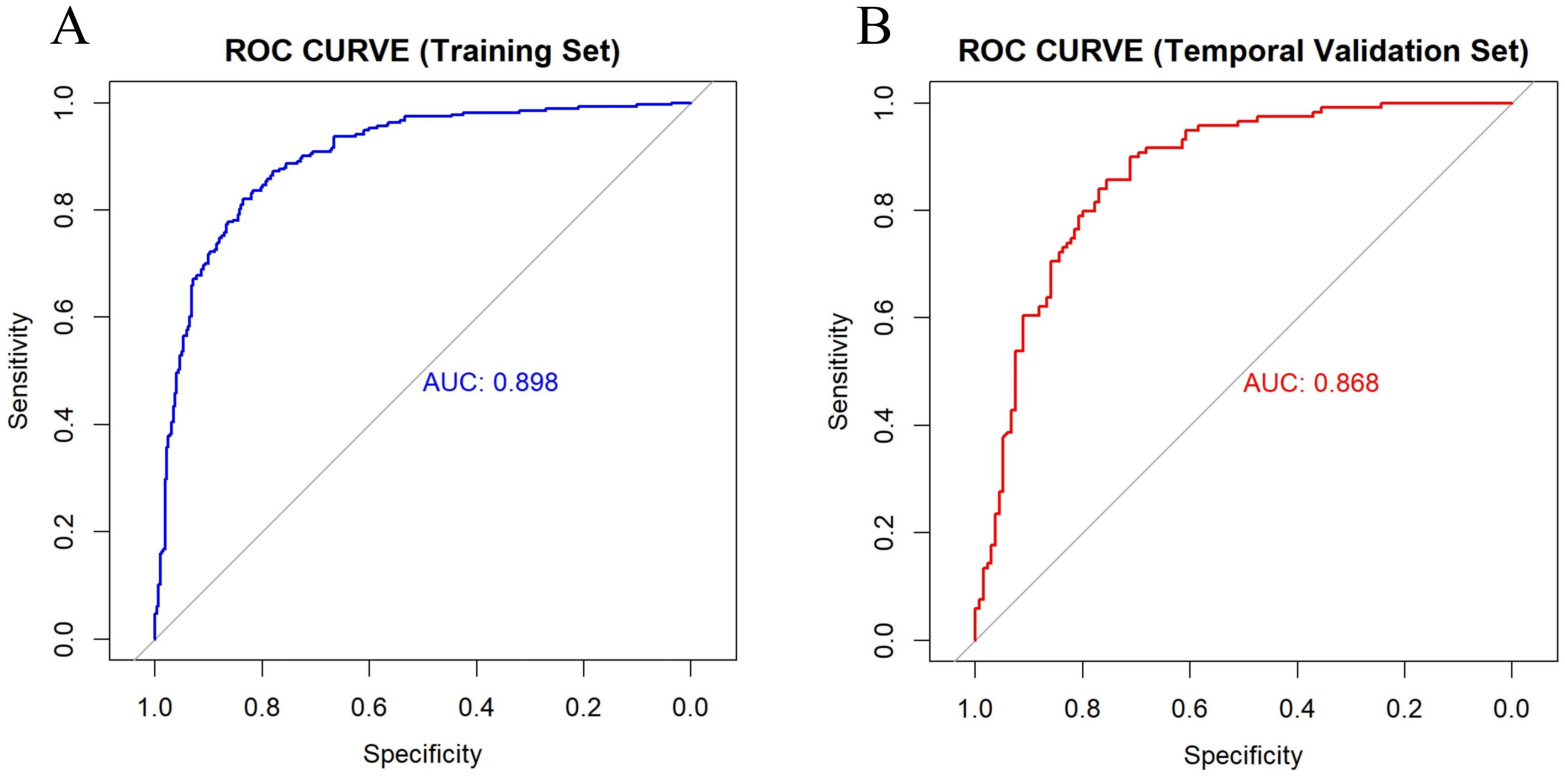
Receiver operating characteristic (ROC) curves evaluating the discriminatory performance of the prediction model. **(A)** ROC curve for the training set (AUC = 0.898). **(B)** ROC curve for the temporal validation set (AUC = 0.868).

For the training group, ROC curve analysis demonstrated an area under the curve (AUC) of 0.898 (95% CI, 0.873–0.923), indicating that the model possesses highly favorable discrimination (Figure 3A). The calibration curve closely approximated the ideal diagonal line (*p* = 0.533), suggesting that the model has high consistency and good calibration (Figure 3B). Furthermore, internal validation using a 1,000-iteration Bootstrap resampling procedure revealed an extremely small optimism bias of 0.0061, yielding an optimism-corrected AUC of 0.8919. This minimal penalization confirms the robustness of the model and indicates a low risk of overfitting.

#### 3.4.3 Temporal validation

**Figure 4:**
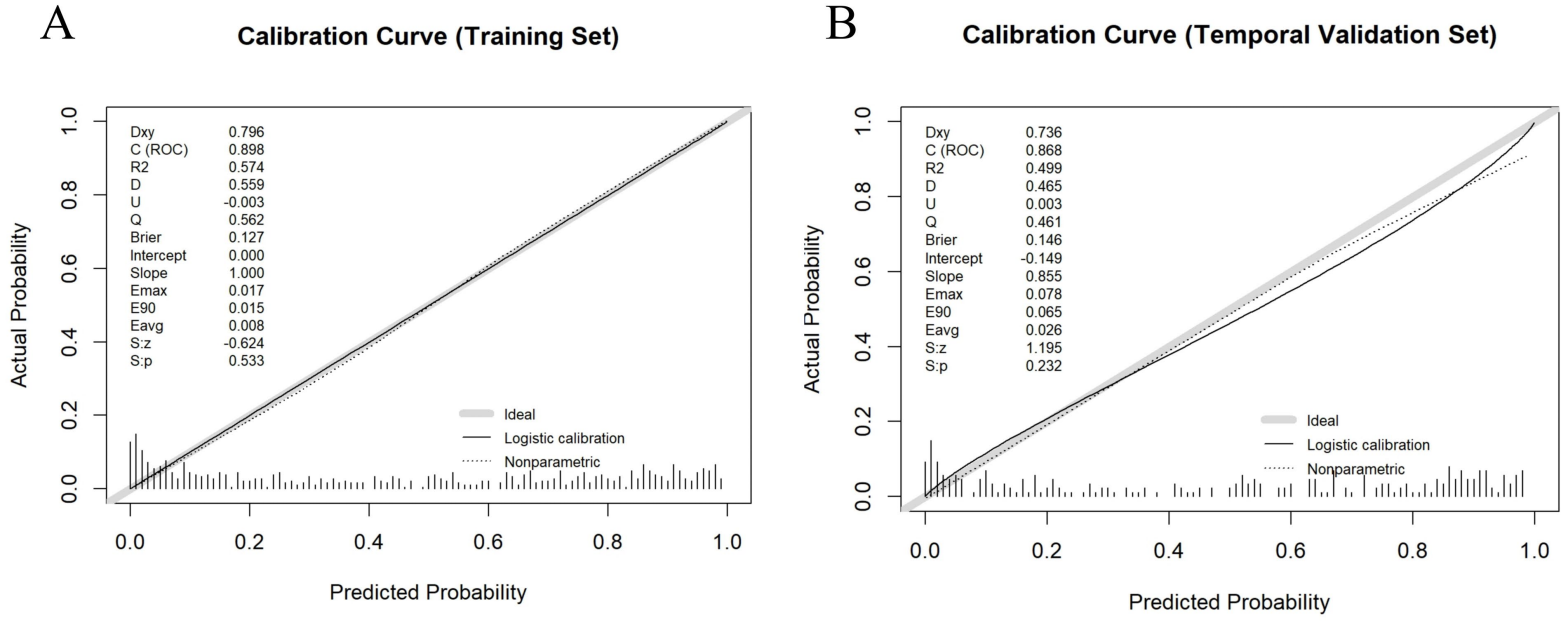
Calibration curves evaluating the consistency between predicted and actual probabilities of Demoralization Syndrome. **(A)** Calibration curve for the training set. **(B)** Calibration curve for the temporal validation set.

For the temporal validation group, ROC curve analysis demonstrated an AUC of 0.868 (95% CI: 0.823–0.912), affirming that the model maintains robust discriminatory ability in subsequent patient cohorts (Figure 4A). The plotted calibration curve also closely approximated the ideal diagonal line (*p* = 0.232), demonstrating good calibration and the high consistency of the model across different timeframes (Figure 4B).

#### 3.4.4 Decision curve analysis

**Figure 5:**
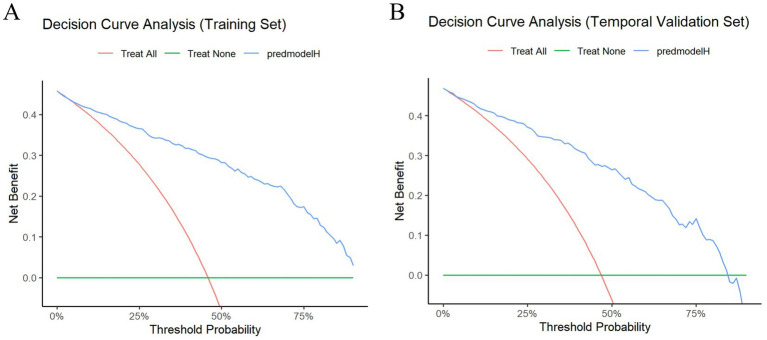
Decision curve analysis (DCA) of the risk prediction model. **(A)** Decision curve for the training set. **(B)** Decision curve for the temporal validation set.

Decision curve analysis (DCA) was conducted to evaluate the potential clinical applicability of the nomogram. The results indicated that utilizing the developed nomogram to predict DS risk provided a higher net clinical benefit compared to the “treat-all” or “treat-none” strategies across a range of threshold probabilities in both the training (Figure 5A) and temporal validation sets (Figure 5B). These findings suggest that the nomogram could serve as a useful reference for clinical nursing decision-making.

## 4 Discussion

This study first screened potential risk factors associated with DS in patients with T2DM to develop a risk prediction model. The findings indicated that T2DM patients in the training group had a mean DS score of 12.01 ± 5.52, suggesting a moderate level of demoralization. Notably, the prevalence of DS reached 45.8%, surpassing the severe demoralization rates among chronic disease patients reported by Dischinger et al. (39). Such a high prevalence may be driven by the complex and rigorous lifelong treatment and self-care routines required for T2DM (e.g., long-term dietary restrictions, blood glucose monitoring, and insulin injections), the constant threat of disabling or fatal acute and chronic complications (e.g., diabetic ketoacidosis, macrovascular and microvascular complications), and the substantial financial burden of healthcare. As the American Diabetes Association (ADA) emphasizes, “Mental health is foundational to achieving diabetes treatment goals” (40).

Therefore, assessing patients’ psychological well-being is crucial as part of diabetes management, and identifying demoralization and reversing negative emotions are vital issues in diabetic mental health care. The risk prediction model for DS in patients with T2DM developed in this study demonstrates favorable overall predictive performance, providing a valuable reference and evidence base for medical staff in the assessment of DS. Furthermore, this study reveals that, ranked by predictive probability, resilience, 2 h PG, confrontation coping style (MCMQ), HbA1c, sleep quality, avoidance coping style (MCMQ), self-management, and educational level are independent predictors for DS in patients with T2DM.

Resilience refers to an individual’s capacity to maintain an optimistic and positive psychological state in the face of adversity and stress. It remains relatively stable regardless of external circumstances and serves to counterbalance negative emotions (20). The present study identifies resilience as a critical predictor of DS among patients with T2DM, a finding consistent with previous research (41). This negative association may be explained by resilience acting as a psychological buffer against “subjective incompetence.” Specifically, individuals with higher resilience are more likely to maintain a clear cognitive map to navigate their illness, helping them avoid hopelessness and distress (42). Furthermore, resilience does not merely act independently; it also moderates the impact of external stressors on mental health by enhancing the protective effects of resources like social and family support (41). This structural negative association between resilience traits and DS has also been corroborated by network analyses in the general population (43). Given that DS is driven by a collapse in coping ability rather than the anhedonia typical of depression, clinical attention to resilience is essential. Healthcare providers can implement Meaning-Centered Psychotherapy (MCP), such as the Kibo therapeutic interview, to assist patients in exploring personal values, reconstructing their life narratives, and directly alleviating the loss of meaning (44). Concurrently, formal cognitive-behavioral programs like Mindfulness-Based Cognitive Therapy (MBCT) should be utilized to challenge automatic thoughts that generate a sense of helplessness, rebuilding the patient’s sense of control (42).

Levels of 2hPG and HbA1c reflect acute postprandial glucose fluctuations and chronic glycemic exposure, respectively (45). Extensive epidemiological data bidirectional dose–response relationship between glycemic dysregulation and severe psychological distress sharing DS phenotypes (46–48). Longitudinally, psychological burden exacerbates glycemic variability, creating a vicious cycle of deteriorating glycemic trajectories and worsening demoralization (49, 50). Mechanistically, chronic hyperglycemia and acute postprandial excursions (2hPG) precipitate low-grade neuroinflammation, compromising prefrontal-striatal motivational circuits and providing a physiological substrate for subjective incompetence (51). To interrupt this pathological loop, clinicians can employ Cognitive Behavioral Therapy (CBT) to help patients reframe transient glucose spikes as actionable metrics rather than personal inadequacies (52). Furthermore, lifestyle modifications, including increased physical activity and optimized sleep, can synchronously enhance insulin sensitivity and psychological well-being (53).

Medical coping modes reflect an individual’s psychological state when addressing challenges. They function as a crucial moderating variable affecting mental health and exert a significant impact on the progression of DS (54). Confrontation coping exerts protective effects by enhancing self-efficacy, prompting proactive disease management and improving metabolic control. This positive feedback reinforces patient agency and buffers existential despair (5). Conversely, avoidance coping (19) provides transient emotional relief but fundamentally disrupts long-term self-management. This initiates a vicious cycle where neglected care accelerates metabolic decline, further amplifying distress and entrenching the subjective incompetence central to DS (55). Healthcare providers can utilize Motivational Interviewing (MI) to resolve patient ambivalence and catalyze the behavioral shift from avoidant to proactive coping (56). Furthermore, integrating Problem-Solving Therapy (PST) allows clinicians to assist patients in dismantling catastrophic illness perceptions and establishing attainable micro-goals (57).

Patients with T2DM generally experience poor sleep quality due to factors such as blood glucose fluctuations, peripheral neuropathy, frequent urination, and adverse medication effects. Research indicates that sleep disturbance serves as a transdiagnostic mechanism in psychopathology, potentially linking to the etiology of various psychiatric conditions through multiple neurobiological pathways, including HPA-axis hyperactivity and systemic inflammation (58). The findings by Vehling et al. (59) demonstrated that patients with poorer sleep quality exhibit elevated levels of DS. Conversely, the existential anxiety and sense of meaninglessness characteristic of DS may contribute to sleep onset and maintenance difficulties, as patients often experience nocturnal rumination regarding the negative consequences of their illness. Clinical practice should prioritize structured aerobic and resistance training to facilitate sleep optimization and neurochemical regulation (60). Furthermore, beyond standard sleep hygiene education, physiological-specific environmental optimization tailored to manage T2DM-related metabolic fluctuations, is essential for sustaining restorative sleep (61).

Self-management capacity emerged as a significant predictor of DS in our study, with lower capacity correlating with heightened demoralization. These findings align with Li et al. (62), who identified self-management skills as a foundational factor in navigating the physical and emotional demands of chronic illness. Furthermore, this relationship is mediated by complex psychological pathways, particularly the interplay between diabetes distress and self-efficacy (63, 64). When self-management capacity is inadequate, persistent glycemic instability undermines self-efficacy, potentially trapping patients in a cycle of learned helplessness and psychological distress. Consequently, clinical practice should transition from perfunctory education to targeted interventions that enhance health literacy and personalized self-management training. By leveraging data-driven feedback models, clinicians can reinforce patient agency and effectively buffer against the transition to DS (65).

Finally, educational attainment is negatively correlated with the severity of DS, a finding consistent with the research by Nanni et al. (66). This correlation may stem from the fact that T2DM patients with higher educational backgrounds possess stronger cognitive comprehension, enabling them to assimilate diabetes self-management information more rapidly. Conversely, patients with lower educational levels may experience psychological distress when confronted with the need to acquire extensive knowledge and master technical procedures. This stress can lead to a refusal to actively engage in learning, thereby allowing the disease to progress unchecked and precipitating a vicious cycle.

In conclusion, the risk prediction model developed in this study for DS among patients with T2DM demonstrates robust overall predictive performance. It provides a reliable reference and solid evidence base for healthcare professionals in the clinical assessment of this syndrome. Within clinical nursing practice, healthcare providers should implement individualized nursing interventions to prevent the onset of DS in T2DM patients, thereby advancing precision medicine, averting adverse events, and alleviating the burden on families.

## 5 Conclusion

Through univariate and binary logistic regression analyses of the clinical data from 598 patients with T2DM, this study identified 8 independent risk factors (resilience, 2 h PG, confrontation coping style (MCMQ), HbA1c, sleep quality, avoidance coping style (MCMQ), self-management, and educational level). A risk prediction model was subsequently developed based on these factors, evaluated, and validated. The results indicated that the model exhibits excellent discrimination and calibration. As a straightforward predictive tool with robust predictive performance, it offers clinical evidence and a practical reference for medical personnel to conduct early assessment and identification, ultimately aiding in the prevention of demoralization syndrome.

## 6 Limitations

Several limitations of this study warrant consideration. First, the recruitment was restricted to a single tertiary hospital in Hefei, Anhui Province. This single-center design potentially introduces selection bias, as the sociodemographic and clinical characteristics of this cohort may not fully generalize to populations in other regions, varying healthcare levels, or primary care settings.

Second, our study utilized a temporal validation strategy within a single center. The lack of an external, multi-center validation cohort means that the generalizability of the model to broader clinical settings remains to be established. Therefore, future large-scale, multi-center studies are essential to rigorously validate and, if necessary, recalibrate the model to ensure its robustness before wide-scale clinical adoption.

Finally, the cross-sectional nature of this study precludes the determination of causal relationships between DS and the identified risk factors. While associations were observed, the temporal precedence of these factors relative to DS cannot be definitively established. Consequently, future longitudinal cohort studies are warranted to further elucidate the complex causal pathways and dynamic transitions among these variables over time.

## Data Availability

The original contributions presented in the study are included in the article/Supplementary material, further inquiries can be directed to the corresponding author.
