# Supplementary material for: Development and validation of a risk prediction model for demoralization syndrome in patients with type 2 diabetes
Source: Front Public Health. 2026 Jul 9;14:1842877. doi: 10.3389/fpubh.2026.1842877 (PMC13391272; doi:10.3389/fpubh.2026.1842877)
Supplement: Supplementary file 1 [file Table_1.DOCX]

Supplementary Material

## Supplementary Tables

## Supplementary Tables 1.Comparison of variable characteristics between the training group and validation group

| Variable | Training group (n=598) | Validation group (n=254) | Statistical value | *P* |
| --- | --- | --- | --- | --- |
| Sex [(n)%] | | | 1.248^a^ | 0.264 |
| Male | 345(57.7) | 157(61.8) |  |  |
| Female | 253(42.3) | 97(38.2) |  |  |
| Ethnicity [(n)%] | | | 1.426^a^ | 0.232 |
| Han Majority | 570(95.3) | 237(93.3) |  |  |
| Ethnic Minorities | 28(4.7) | 17(6.7) |  |  |
| Age [years old, (n)%] | | | 0.021^a^ | 0.884 |
| ＜45 | 119(19.9) | 50(19.7) |  |  |
| 45~60 | 219(36.6) | 92(36.2) |  |  |
| ＞60 | 260(43.5) | 112(44.1) |  |  |
| Educational Level [(n)%] | | | 0.004^a^ | 0.951 |
| Uneducated | 75(12.5) | 33(13.0) |  |  |
| Elementary School | 114(19.1) | 48(18.9) |  |  |
| Junior high School | 107(17.9) | 45(17.7) |  |  |
| High School | 129(21.6) | 54(21.3) |  |  |
| Associate Degree or Higher | 173(28.9) | 74(29.1) |  |  |
| Marital Status [(n)%] | | | 1.242^a^ | 0.537 |
| Unmarried | 28(5.4) | 9(3.5) |  |  |
| Married | 518(86.6) | 227(89.4) |  |  |
| Divorced or Widowed | 52(8.7) | 18(7.1) |  |  |
| Residence [(n)%] | | | 0.113^a^ | 0.737 |
| Town | 386(64.5) | 167(65.7) |  |  |
| Rural | 212(35.5) | 87(34.3) |  |  |
| Work Difficulty [(n)%] |  |  | 0.343^a^ | 0.558 |
| Easy | 151(25.3) | 67(26.4) |  |  |
| Moderate | 272(45.5) | 118(46.5) |  |  |
| Difficult | 175(29.3) | 69(27.2) |  |  |
| Per Capita Monthly Household Income [(RMB), (n)%] | | | 1.836^a^ | 0.175 |
| ＜1000 | 61(10.2) | 33(13.0) |  |  |
| 1000~3000 | 126(21.1) | 57(22.4) |  |  |
| 3000~5000 | 204(34.1) | 84(33.1) |  |  |
| ＞5000 | 207(34.6) | 80(31.5) |  |  |
| Medical Payment Method [(n)%] | | | 0.219^a^ | 0.896 |
| Employee Medical Insurance | 325(54.3) | 134(52.8) |  |  |
| Urban Resident Medical Insurance | 254(42.5) | 111(43.7) |  |  |
| Self-funded | 19(3.2) | 9(3.5) |  |  |
| Family Care Methods [(n)%] | | | 0.375^a^ | 0.540 |
| Family Involvement in Management Assistance | 290(48.5) | 129(50.8) |  |  |
| No Family Involvement in Management Assistance | 308(51.5) | 125(49.2) |  |  |
| Smoking History [(n)%] |  |  | 0.163^a^ | 0.687 |
| Yes | 187(31.3) | 83(32.7) |  |  |
| No | 411(68.7) | 171(67.3) |  |  |
| Drinking History [(n)%] |  |  | 0.697^a^ | 0.404 |
| Yes | 169(28.3) | 79(31.1) |  |  |
| No | 429(71.7) | 175(68.9) |  |  |
| BMI[Kg/m², (n)%] | | | 4.254^a^ | 0.039 |
| ＜18.5 | 15(2.5) | 1(0.4) |  |  |
| 18.5~ | 198(33.1) | 72(28.3) |  |  |
| 24~ | 238(39.8) | 110(43.3) |  |  |
| ＞28 | 147(24.6) | 71(28.0) |  |  |
| Diabetes Course[year, (n)%] | | | 0.009^a^ | 0.924 |
| ＜1 | 93(15.6) | 44(17.3) |  |  |
| 1-5 | 135(22.6) | 48(18.9) |  |  |
| 6-10 | 108(18.1) | 49(19.3) |  |  |
| ＞10 | 262(43.8) | 113(44.5) |  |  |
| Diabetes Complications[(n)%] | | | 0.227^a^ | 0.634 |
| No | 277(46.3) | 117(46.1) |  |  |
| 1 | 154(25.8) | 60(23.6) |  |  |
| 2 | 96(16.1) | 44(17.3) |  |  |
| ≥3 | 71(11.9) | 33(13.0) |  |  |
| FBG [mmol/L，($\bar{\text{x}}\text{±}\text{s}$)] | 8.45±2.78 | 8.24±2.61 | 1.093^b^ | 0.296 |
| 2h PG [mmol/L，($\bar{\text{x}}\text{±}\text{s}$)] | 13.17±4.78 | 13.64±4.98 | 1.645^b^ | 0.200 |
| HbA1c [%，($\bar{\text{x}}\text{±}\text{s}$)] | 9.94±3.26 | 10.00±3.23 | 0.071^b^ | 0.790 |
| TCHoL [mmol/L，($\bar{\text{x}}\text{±}\text{s}$)] | 3.63±1.99 | 3.39±1.82 | 2.853^b^ | 0.091 |
| TG [mmol/L, ($\bar{\text{x}}\text{±}\text{s}$)] | 2.95±2.44 | 2.99±2.41 | 0.062^b^ | 0.804 |
| HDLC [mmol/L, ($\bar{\text{x}}\text{±}\text{s}$)] | 1.48±0.61 | 1.45±0.61 | 0.345^b^ | 0.557 |
| LDLC [mmol/L, ($\bar{\text{x}}\text{±}\text{s}$)] | 3.79±1.78 | 3.81±1.86 | 0.018^b^ | 0.892 |
| HCY [μmol/L, ($\bar{\text{x}}\text{±}\text{s}$)] | 12.66±6.35 | 12.51±6.69 | 0.099^b^ | 0.753 |
| GA [%, ($\bar{\text{x}}\text{±}\text{s}$)] | 21.95±7.04 | 21.26±6.10 | 1.779^b^ | 0.182 |
| FINS [mU/L, ($\bar{\text{x}}\text{±}\text{s}$)] | 14.66±7.23 | 14.39±7.11 | 0.251^b^ | 0.616 |
| FCP [pmol/L, ($\bar{\text{x}}\text{±}\text{s}$)] | 2.65±1.40 | 2.75±1.38 | 0.823^b^ | 0.364 |
| SSRS [score, ($\bar{\text{x}}\text{±s}$)] | 26.05±3.67 | 26.17±3.72 | 0.165^b^ | 0.684 |
| MCMQ [score, ($\bar{\text{x}}\text{±s}$)] |  |  |  |  |
| Confrontation | 19.05±3.29 | 18.86±3.10 | 0.580^b^ | 0.446 |
| Avoidance | 17.49±2.62 | 17.49±2.64 | 0.000^b^ | 0.986 |
| Resignation | 11.80±2.61 | 11.76±2.64 | 0.041^b^ | 0.840 |
| SDSCA [score, ($\bar{\text{x}}\text{±s}$)] | 23.25±8.28 | 22.45±8.02 | 1.694^b^ | 0.193 |
| CD-RISC [score, ($\bar{\text{x}}\text{±s}$)] | 41.08±10.30 | 40.72±10.228 | 0.221^b^ | 0.638 |
| PSQI [score, ($\bar{\text{x}}\text{±s}$)] | 8.10±3.10 | 8.11±2.90 | 0.002^b^ | 0.960 |

^a^ χ^2^value；^b^ *t* value.

Abbreviations: FBG, Fasting blood glucose;

2h PG, two-hour postprandial plasma glucose;

HbAlc, Glycated Hemoglobin A1c;

TCHoL, total cholesterol;

TG, Triglycerides;

HDLC, high-density lipoprotein cholesterol;

LDLC, Low-Density Lipoprotein Cholesterol;

HCY, Homocysteine;

GA, glycated albumin;

FINS, Fasting Insulin;

FCP, Fasting C-Peptide;

SSRS, social support rating scale;

SDSCA, Summary of Diabetes Self-Care Activities;

MAMQ, Medical Coping Modes Questionnaire;

CD-RISC, Connor Davidson Resilience Scale;

PSQI, Pittsburgh Sleep Quality Index.

## Supplementary Table S2. Variable assignment of predictors in the final model

| Variable | Variable assignment |
| --- | --- |
| Educational Level | Uneducated=1, Elementary School=2, Junior High School=3, High School=4, Associate Degree or Higher=5 |
| 2h PG | Original value |
| HbA1c | Original value |
| SDSCA score | Original value |
| MCMQ score |  |
| Confrontation | Original value |
| Avoidance | Original value |
| CD-RISC score | Original value |
| PSQI score | Original value |

Abbreviations: 2h PG, two-hour postprandial plasma glucose;

HbAlc, Glycated Hemoglobin A1c;

SDSCA, Summary of Diabetes Self-Care Activities;

MAMQ, Medical Coping Modes Questionnaire;

CD-RISC, Connor Davidson Resilience Scale;

PSQI, Pittsburgh Sleep Quality Index.
